# Intestinal Microbiota of *Anser fabalis* Wintering in Two Lakes in the Middle and Lower Yangtze River Floodplain

**DOI:** 10.3390/ani13040707

**Published:** 2023-02-17

**Authors:** Kai Zhao, Duoqi Zhou, Mengrui Ge, Yixun Zhang, Wenhui Li, Yu Han, Guangyu He, Shuiqin Shi

**Affiliations:** Anhui Key Laboratory of Biodiversity Research and Ecological Protection in Southwest Anhui, School of Life Sciences, Anqing Normal University, Anqing 246133, China

**Keywords:** 16S rRNA amplicon sequencing, *Anser fabalis*, habitat size, intestinal microbiota, wintering waterbirds

## Abstract

**Simple Summary:**

*A. fabalis* are the main migratory waterfowl during the winter. The main habitats of A. fabalis are Caizi lake and Shengjin Lake in the Middle and Lower Yangtze River Floodplain. We utilized high-throughput sequencing and bioinformatics to analyze the composition and structural characteristics of the intestinal microbiota of Caizi Lake (CL) and Shengjin Lake (SL) *A. fabalis* during overwintering. Lactobacillus is the main genus of *A. fabalis*, of which *Lactobacillus aviaries* is the highest. There was only a positive correlation between Bacteroidetes and Proteobacteria in the intestinal microbiota of SL *A. fabalis*, and the species were closely related. However, CL is mainly associated with a positive correlation between Firmicutes and Actinomycetes. The functions of intestinal microbiota of *A. fabalis* involve energy production and transformation, amino acid transport and metabolism, carbohydrate transport and metabolism, and transcription. This study provides basic data for the study of intestinal microbiota of *A. fabalis*.

**Abstract:**

The intestinal microbiota of migratory birds participate in the life activities of the host and are affected by external environmental factors. The difference in habitat environment provides diversity in external environmental selection pressure for the same overwintering waterfowl, which may be reflected in their intestinal microbiota. Caizi lake and Shengjin Lake in the Middle and Lower Yangtze River Floodplain are the main habitats for migratory waterfowl in winter, especially the *Anser fabalis* (*A. fabalis*). It is important to explore the changes in intestinal microbiota composition and function of *A. fabalis* in the early overwintering period to clarify the effect of habitat size and protection status on intestinal microbiota. In this study, the composition and structural characteristics of the intestinal microbiota of *A. fabalis* in Shengjin Lake (SL) and Caizi Lake (CL) were preliminarily explored in order to obtain data for the migratory birds. In both SL and CL groups, 16S rRNA amplicon sequencing analysis showed that Firmicutes was the dominant bacterial phylum, but the relative abundance showed significant differences. *Lactobacillus* was the most abundant genus in both SL and CL groups. At the species level, the abundance of *L. aviaries* was the highest, with a relative abundance in both SL and CL groups of more than 34%. When comparing the average relative abundance of the 15 most abundant genera, it was found that *Subdoligranulum*, *Exiguobacterium*, and *Terrisporobacter* had higher abundances in the intestinal microbiota of CL *A. fabalis*, while *Streptococcus* and *Rothia* had higher abundances in the intestinal microbiota of SL *A. fabalis*. There was only a positive correlation between *Bacteroidota* and *Proteobacteria* in the intestinal microbiota flora of SL *A. fabalis*, and the species were closely related. At the same time, there were positive and negative correlations between Firmicutes and Actinomycetes. However, CL is mainly associated with a positive correlation between Firmicutes and Actinomycetes, and there are also a small number of connections between Firmicutes. PICRUSt1 prediction analysis revealed that the Clusters of Orthologous Groups (COG) functions of SL and CL involve energy production and transformation, amino acid transport and metabolism, carbohydrate transport and metabolism, and transcription. Understanding the changes in intestinal microbiota in Aves during the overwintering period is of great importance to explore the adaptation mechanism of migratory Aves to the overwintering environment. This work provides basic data for an *A. fabalis* intestinal microbiota study.

## 1. Introduction

Bird migration is a regular seasonal movement between breeding sites and wintering sites. Shengjin Lake (SL) and Caizi Lake (CL) in Anhui Province are the main migration protection wetlands for migratory birds. Thousands of migratory birds stay here every year. The first birds of the year come in November and leave in March of the following year. Migratory birds have many unique life history characteristics, such as long-distance travel between breeding sites, stopovers, and wintering sites. During migration, birds need energy [1] and face physiological challenges such as diseases [2], which may lead to changes in intestinal microbiota. Animal microbes are used worldwide in ecotoxicological studies as bioindicator organisms because of their broad distribution, sessile nature, filter-feeding behavior, and tolerance to many pollutants [3]. There are tens of thousands of microbial microbes in the intestines of migratory birds, which form a complex and diverse biological community [4]. The complex network relationship of intestinal microbiota evolves dynamically by diet, environment, and season. The homeostasis of intestinal microbiota plays an important role in host development, nutrient absorption, immune homeostasis, and other physiological functions [5,6]. Intestinal microbiota are important symbionts that affect the life of host birds [7], and the host is the key factor affecting intestinal microbiota [8]. The similarity between intestinal microbiota of the same species is greater than that of different species, and the difference between populations is always significantly greater than that within populations [1]. In addition to being affected by the host itself, the environment is defined as one of the main factors leading to the diversity of animal intestinal microbiota [9,10]. The lake environment has a certain impact on water birds [11]. The water temperature, salinity, and pH of the lake environment have an impact on the organisms living in the habitat [12]. Studies have shown that different species of intestinal microbiota are isolated, but direct or indirect contact in the mixture population will lead to cross-species transmission of intestinal microbiota [13,14]. In particular, this occurs frequently among mixed species with overlapping niches, mainly through sharing the same habitat and food resources to strengthen the relationship between microbial communities and cross-species horizontal transmission of bacteria [14]. This also promotes the spread of pathogenic bacteria among different hosts and increases the difficulty of preventing and controlling infectious diseases. Compared with other birds, there is still less research on wild waterfowl, and there is little understanding of the intestinal microbiota of the same species from different geographical populations, especially in the intestinal microbiota of *A. fabalis*, which migrates over long distances during the overwintering period.

*A. fabalis* is a member of the Anatidae family (order Anseriformes) and an important wetland indicator species [11]. In China, *A. fabalis* mainly stays in SL and CL in the middle and lower reaches of the Yangtze River for the winter (from late October of each year to the middle of March of the following year). In CL, underground tubers are mainly used, while SL also uses gramineous plants as a food source. Studies have shown that this difference in food sources affects the composition and structure of intestinal microbiota of overwintering white-headed geese [15,16]. Studies have shown that direct or indirect contact between hooded cranes and bean geese with overlap at in Shengjin Lake, China, increases the diversity of host intestinal microbiota, and causes bacteria to spread among species in the mixed population [10]. Understanding the composition and characteristics of intestinal microbiota is of great significance to the protection of migratory birds.

We used high-throughput sequencing and bioinformatics to analyze the composition and structural characteristics of the intestinal microbiota of CL and SL *A. fabalis* during overwintering. At the same time, combined with PICRUSt1 (https://picrust.github.io/picrust/install.html#install (accessed on 1 January 2023)), we predicted the intestinal microbiota function of CL and SL *A. fabalis*, and preliminarily analyzed the intestinal microbiota function of *A. fabalis* in different habitat areas, so as to provide basic data for the study of intestinal microbiota of *A. fabalis*.

## 2. Materials and Methods

### 2.1. Study Areas

**Figure 1:**
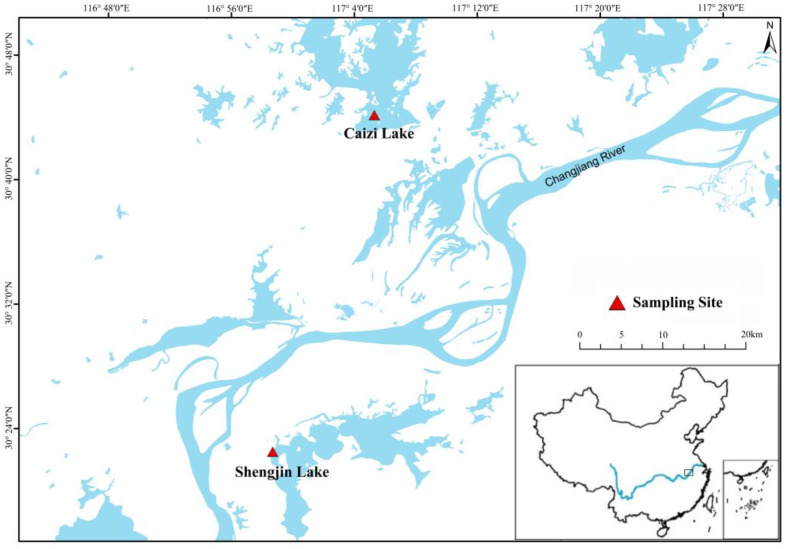
Sampling distribution diagram.

The study was conducted at Caizi (117°2′26.899″ E, 30°44′10.192″ N) and Shengjin (116°58′49.023″ E, 30°22′30.6″ N) Lakes, located in the middle and lower Yangtze River floodplain (Figure 1). Both lakes are important wintering and stopover habitats for migratory wading birds on the East Asia Australasia flight route [17]. Caizi Lake and Shengjin Lake are shallow lakes connected by rivers, belonging to the northern subtropical monsoon climate. Caizi Lake has a water level of 11.00 m, a length of 25.7 km, a maximum width of 7.9 km, an average width of 6.7 km, an area of 172.1 km^2^, a maximum water depth of 8.28 m, an average water depth of 1.67 m, and a water storage capacity of 2.87 × 10^8^ m^3^. The lake area has a northern subtropical monsoon climate, with an average annual temperature of 16.5 °C, an average temperature of 3.6 °C in January, an extreme minimum temperature of −13.5 °C (5 February 1969), an average temperature of 28.7 °C in July, and an extreme maximum temperature of 40.9 °C (23 August 1954). The annual average sunshine duration is 2064.9 h, and the total radiation is 484,629 J/cm^2^, with a frost-free period of 315 days. The precipitation is 1241.3 mm, the maximum annual precipitation is 2266.1 mm (1954), and the minimum annual precipitation is 687.6 mm (1978). The catchment area is 3234.0 km^2^, and the recharge coefficient is 18.8. Shengjin Lake is a reservoir type with a water level of 11.00 m, a length of 20.5 km, a maximum width of 7.5 km, and an average width of 3.83 km^2^. The original area is 146.7 km^2^, and the area after reclamation is 78.48 km^2^, including 32.14 km^2^ for the upper lake, 37.64 km^2^ for the middle lake, and 8.7 km^2^ for the lower lake. The maximum water depth is 3.50 m, the average water depth is 1.26 m, and the storage capacity is 0.99 × 10 m^3^. The lake area has a northern subtropical monsoon climate, with an average annual temperature of 16.1 °C, an average temperature of 3.1 °C in January, an extreme minimum temperature of −15.6 °C (5 February 1960), an average temperature of 28.5 °C in July, and an extreme maximum temperature of 40.6 °C (August 1971). The average annual sunshine duration is 1474.3 h, and the frost-free period is 223 days. The precipitation is 1554.4 mm, the maximum annual precipitation is 2183.2 mm (1954), and the minimum annual precipitation is 1015.2 mm (1978); The average annual Meiyu is 332.5 mm, the maximum annual Meiyu is 863.7 mm (15 June–19 July 1969), and the minimum annual Meiyu is 58.1 mm (11–26 June 1958). The evaporation capacity is 1556 mm. The catchment area is 1554.0 km^2^, and the recharge coefficient is 19.8.

### 2.2. Sample Collection

Based on a preliminary study of bird flight trajectories by our research group, the yang’e head of Shengjin Lake and Plum Blossom Dawei of Caizi Lake were selected as the experimental sample collection points. Before collecting samples, we selected a large population of more than 300 *Anser fabalis* at the specific locations using binoculars. We ensured that the *A. fabalis* entered the foraging area for feces collection after foraging for more than two hours. In order to avoid human interference and soil pollution, we observed *A. fabalis* to collect fresh fecal samples immediately after foraging and defecation. At the same time, in order to avoid the repeated collection of the same *A. fabalis* sample as much as possible, we ensured that the distance between samples was more than 5 m [18], and each sample was not less than 2 g. Every 10 samples were mixed into one fecal sample, and 60 mixed samples were collected (Table 1). All samples were collected from the center of each fecal block and then quickly put into a sterile EP tube, frozen in liquid nitrogen, transported to the laboratory in liquid nitrogen, and stored at −80 °C.

### 2.3. DNA Extraction and Sequencing

Microbial DNA was extracted with the QIAamp Fast DNA Stool Mini Kit. The final DNA concentration and purity were determined by nanodrop 2000 UV-Vis spectrophotometer (Thermo Fisher Scientific, Waltham, MA, USA). The optical density ratio of OD260/280 = 1.8–2.0 indicated optimal DNA purity. The V3–V4 hypervariable regions of the bacterial 16S rRNA gene were amplified with primers 338F (5′-ACTCCTACGGGAGGCAGCAG-3′) and 806R (5′-GGACTACHVGGGTWTCTAAT-3′) on a PCR thermocycler system, further purified with an AxyPrep DNA Gel Extraction Kit (Axygen Biosciences, Union City, CA, USA), and quantified with a QuantiFluor™-ST fluorometer (Promega, Madison, WI, USA). Equimolar amounts of the purified amplicons were pooled and paired-end sequenced (2 × 300) on the Illumina MiSeq platform (Illumina, San Diego, CA, USA), with standard protocols (Majorbio Bio-Pharm Technology Co., Ltd., Shanghai, China).

### 2.4. Sequencing Data Analysis

The 16S rRNA data and their richness were investigated with the Quantitative Insights Into Microbial Ecology 1.9.0 software (QIIME; http://qiime.org, accessed on 16 October 2022). The Illumina adapters and primers were removed from the raw sequences. The trimmed forward and reverse sequences were combined [19]. These sequences were clustered into OTUs (97% similarity) with UCLUST [20]. The reference OTU sequences were taxonomically assigned with the UCLUST Consensus Taxon Assigner (DeSantis et al., 2006) against the Green genes database [21], with a 0.5 confidence threshold, and identified to the species level. Rarefied OTUs were used to measure the bacterial richness from the total lengths of the phylogenetic branches [22] and the relative proportions of rare sequences [23]. Based on the sample information, a redundancy analysis with clustered OTUs was used to compare the chicken breed with the bacterial community structures using the R statistical software version 3.3.0 [24]. To assess whether both chicken-breed-specific microbiomes were significantly distinguished, we used a nonparametric statistical test analysis of similarity. The significance of differences between groups was determined with permutations (*n* = 999) using the vegan package in the R statistical software. Using mothur software, co-occurrence analysis among genera was investigated by calculating C-scores, and Spearman’s rank correlations of the 30 most abundant genera were calculated. Network analysis using the genera with rho > 0.6 and *p* < 0.01 was visualized using Cytoscape (version 3.4.0). FAPROTAX software (version 1.2.6) was used to predict the sample functional abundance according to the sample microbial abundance table [25].

### 2.5. Data Deposition

The raw 16S rRNA gene sequences are accessible through Sequence Read Archive (SRA) study accession number SRP370299.

## 3. Results

### 3.1. Comparative Analysis of Intestinal Microbiota Composition of SL and CL A. fabalis

**Figure 2:**
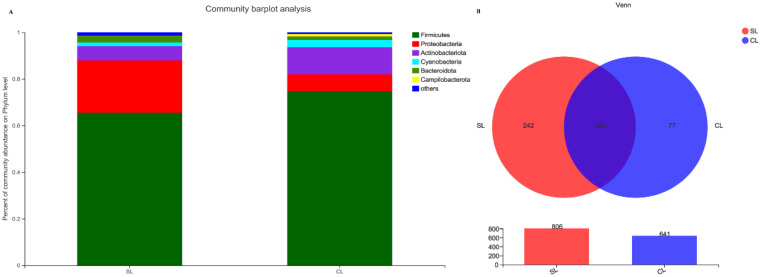
(**A**) represents the composition of intestinal microbiota of *Anser fabalis* at the phylum level; (**B**) shows the Venn diagram of intestinal microbiota in *Anser fabalis* at the genus level.

The sample sequences were classified and annotated, and the intestinal microbiota composition of SL and CL *A. fabalis* were analyzed at the phylum level. It was found that the total number of annotated phyla was the same, but the relative abundance of each phylum showed significant differences (*p* < 0.05). At the phylum level, the intestinal microbiota of *A. fabalis* were Firmicutes (SL: 65.47%; CL: 74.71%), Proteobacteria (SL: 22.49%; CL: 7.28%), Actinobacteriota (SL: 6.13%; CL: 11.60%), Cyanobacteria (SL: 1.57%; CL: 3.14%), and Bacteroidota (SL: 2.83%; CL: 1.50%). The dominant phylum of *A. fabalis* was Firmicutes and Proteobacteria (Figure 2A). We identified 883 genera, 564 of which appeared to be present in all samples, while 242 and 77 were uniquely present in the *A. fabalis* from SL and CL, respectively (Figure 2B).

**Figure 3:**
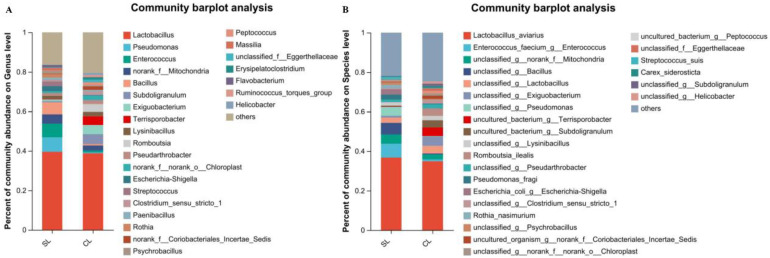
(**A**) shows the composition of intestinal microbiota of *Anser fabalis* at the genus level; (**B**) represents the composition of intestinal microbiota of *Anser fabalis* at the species level.

*Lactobacillus* was the main genus in both SL and CL *A. fabalis*, with a relative abundance of 39.54% and 38.69%, respectively. In SL *A. fabalis*, the relative abundance of *Pseudomonas* was 7.34%, that of *Enterococcus* was 6.99%, and that of *Bacillus* was 6.09%. In CL *A. fabalis*, the relative abundance of *Subdoligranulum* was 5.02%, that of *Exiguobacterium* was 4.68%, and that of *Terrisporobacter* was 4.40% (Figure 3A). At the species level, the species *L. aviaries* was most abundant in both SL and CL *A. fabalis*, with a relative abundance of more than 34%. The annotated species of intestinal microbiota of SL *A. fabalis* were followed by *Enterococcus faecium* (relative abundance 6.99%), *Mitochondria* (relative abundance 4.67%), *Bacillus* (relative abundance 5.92%), *Lactobacillus* (relative abundance 2.70%), and *Pseudomonas* (relative abundance 4.56%). The annotated species of intestinal microbiota of CL *A. fabalis* were followed by *Mitochondria* (relative abundance 2.54%), *Lactobacillus* (relative abundance 3.81%), and *Geosporobacteria* (relative abundance 4.40%) (Figure 3B).

**Figure 4:**
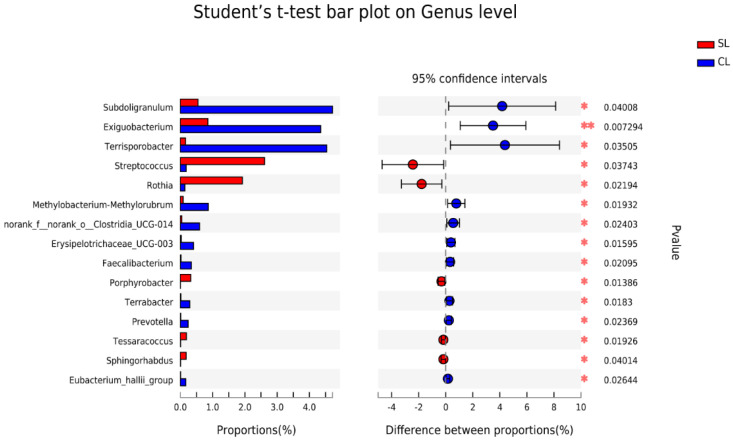
Column chart of intestinal multi—species difference test of SL and CL *Anser fabalis.* The *p* value: * *p* < 0.05; ** *p* < 0.01.

The composition and average relative abundances of the top 15 genera in the intestinal microbiota of SL and CL *A. fabalis* were compared and analyzed. It was found that *Subdoligranulum* (*p* < 0.05), *Exiguobacterium* (*p* < 0.01), and *Terrisporobacter* (*p* < 0.05) had higher abundance in the intestinal microbiota of CL *Anser fabalis*, while *Streptococcus* (*p* < 0.05) and *Rothia* (*p* < 0.05) were more abundant in the intestinal microbiota of SL *A. fabalis* (Figure 4).

### 3.2. Interaction Analysis of Intestinal Microbiota Network of SL and CL A. fabalis

**Figure 5:**
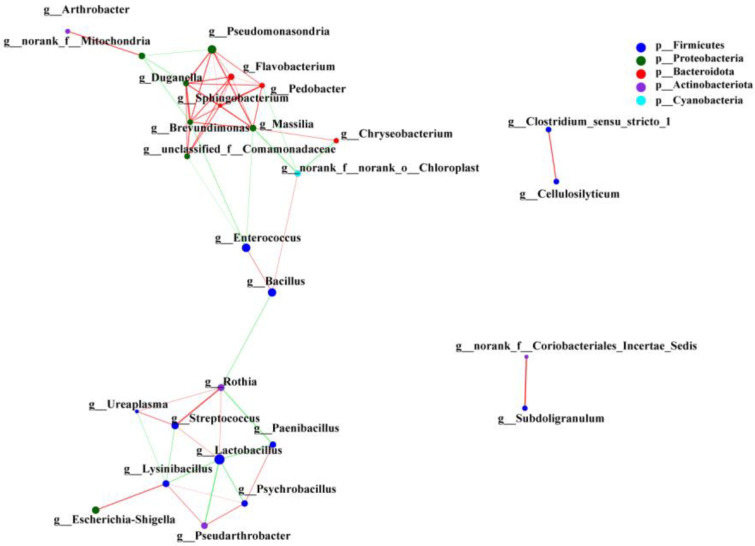
Single factor correlation network analysis of intestinal microbiota of SL *Anser fabalis*.

The Spearman correlation coefficient among the species with a total relative abundance in the top 30 at the genus level in the intestinal microbiota of SL *A. fabalis* was calculated using network analysis. The results showed that there was only a positive correlation between *Proteobacteria* and *Bacteroidota* in the intestinal microbiota of SL *A. fabalis*, and the species were closely related, while there were positive and negative network interactions between *Firmicutes* and *Actinobacteria* (Figure 5).

**Figure 6:**
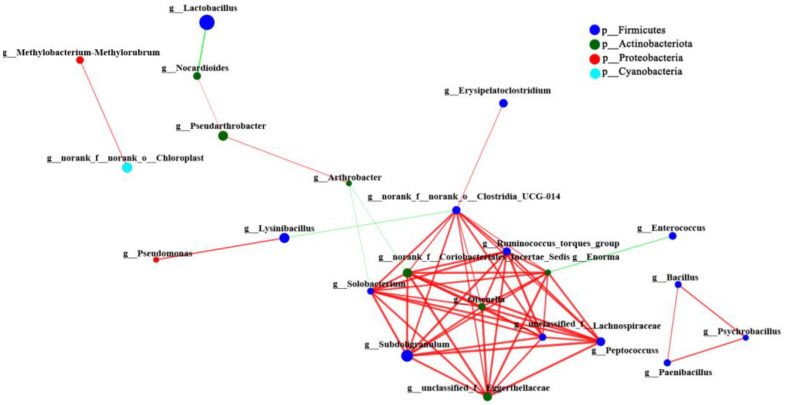
Single factor correlation network analysis of intestinal microbiota of CL *Anser fabalis*.

The Spearman correlation coefficient was calculated between the total abundance of CL *A. fabalis* intestinal microbiota species in the top 30 genera. As shown in Figure 6, the positive correlation between *Firmicutes* and *Actinomycetes* is the main network relationship in the intestinal microbiota of CL *A. fabalis*, and there is also a low correlation with *Firmicutes*.

**Figure 7:**
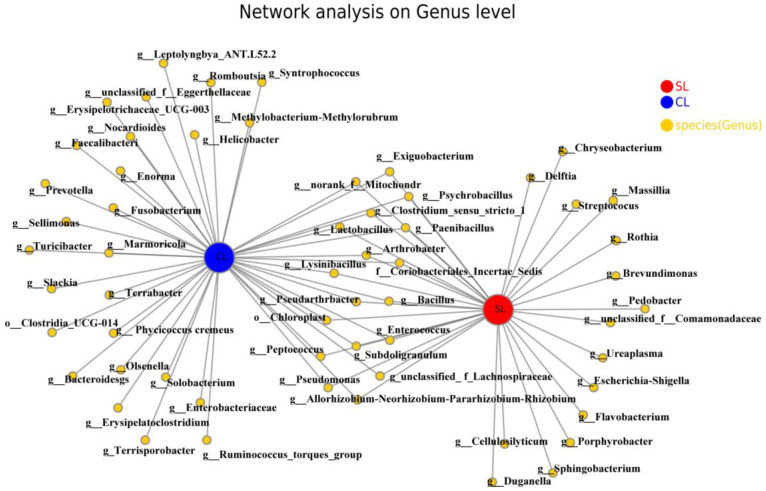
Network analysis of intestinal microbiota of SL and CL *Anser fabalis*.

At the genus level, the network collinearity network analysis was carried out for the intestinal microbiota of SL and CL *A. fabalis*. As shown in Figure 7, the two sample collinear network species have 18 genera.

### 3.3. Functional Comparative Analysis of Intestinal Microbiota Genomes of SL and CL A. fabalis

**Figure 8:**
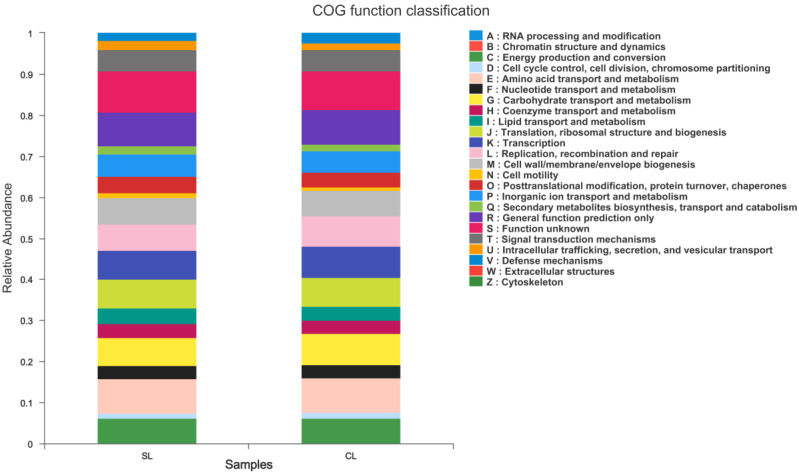
Statistical histogram of COG function classification of intestinal microbiota of SL and CL *Anser fabalis*.

The OTU abundance tables of SL and CL samples were predicted and analyzed by PICRUSt1, and the annotation information of OTUs at the Clusters of Orthologous Group (COG) function level and the abundance information of each function in different samples were obtained. The results showed that there was no significant difference in the relative abundance of annotated COGs between SL and CL groups (*p* > 0.05), mainly including energy production and transformation, amino acid transport and metabolism, carbohydrate transport and metabolism, and transcription (*p* > 0.05; Figure 8).

**Figure 9:**
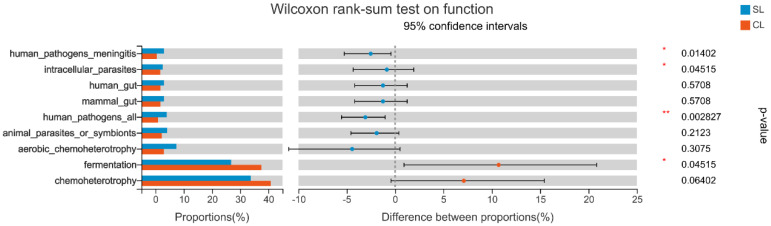
Difference test between functional groups of intestinal microbiota of SL and CL *Anser fabalis.* The *p* value: * *p* < 0.05; ** *p* < 0.01.

At the same time, the differences between functional groups of SL and CL samples were analyzed by FAPROTAX, and the two groups of biological taxa were mapped to analyze metabolism or other ecological-related functions. Figure 9 shows the difference between the two groups of *A. fabalis* intestinal microbiota function predicted by FAPROTAX function (top 9). There were significant differences in the functions of SL and CL *A. fabalis* in human meningitis pathogens, intracellular parasites, and fermentation functions (0.01 < *p* ≤ 0.05), and there were extremely significant differences in their functions as human pathogens (0.001 < *p* ≤ 0.01).

## 4. Discussion

Intestinal microbiota are affected by environment, disease, and other factors, but these factors are rarely reported in overwintering birds. Data annotation analysis showed that the intestinal microbiota of SL and CL *A. fabalis* were composed of six phyla: Firmicutes, Proteobacteria, Actinomycetes, Cyanobacteria, Bacteroidetes, and Campylobacter. The dominant phylum was Firmicutes (relative abundance was greater than 65%), but there was no significant difference between SL and CL (*p* > 0.05).

A previous study used high-throughput sequencing to reveal the core intestinal microbiota of *A. fabalis* in different wintering areas of Tibet. The results showed that the intestinal microbiota of the Bar-headed goose (*A. indicus*) had 14 representative flora, mainly Firmicutes, Proteobacteria, Actinomycetes, and Bacteroidetes [26]. Zhang found that the dominant phyla of migratory birds’ intestinal microbiota were mainly Firmicutes, Actinomycetes, Proteobacteria, Bacteroidetes, and Clostridium [26]. Wang found that Firmicutes, Proteus, Actinomyces, and Bacteroides are the four most abundant bacteria in the intestines of the Bantou Goose and that carbohydrate, amino acid, nucleotide, and energy metabolism in their microbiomes are the four most abundant [27]. In the annotation analysis at the genus and species levels, we found that *Lactobacillus* was the main genus of *A. fabalis* in SL and CL, and *L. aviaries* was the main strain, with a relative abundance of more than 34% (*p* > 0.05 between SL and CL). The relative abundance of genera and species of lactic acid bacteria was the highest in the SL and CL samples. Some studies have shown that the composition of intestinal microbiota of gray geese is consistent with the main composition in this study, but there are differences in relative abundance composition [28]. There were significant differences among secondary species such as *E. faecium*, *Bacillus*, and *Pseudomonas* (*p* < 0.01). The obvious differentiation of intestinal microbiota structure may indicate that the intestinal microbiota of *A. fabalis* are highly sensitive to the food sources of the two lakes [6]. SL and CL provide abundant and diverse food sources for *A. fabalis* during overwintering [25]. Like other waterfowl species, *A. fabalis* may ingest foods with high starch content, such as the tubers of bitter grass and the grains of surrounding fields, which may increase the relative abundance of Firmicutes in the intestine of *A. fabalis*. Studies have shown that Firmicutes is the dominant bacterium in vertebrates, and it participates in nutrient absorption and energy acquisition. This is related to its function in sugar, carbohydrate, and fatty acid decomposition, and the energy it produces can be used by the host [29].

Univariate correlation network analysis revealed that there was only a positive correlation between *Bacteroidetes* and *Proteobacteria* in the intestinal microbiota of SL *A. fabalis*, and the flora were closely related. At the same time, there were positive and negative correlations between *Firmicutes* and *Actinomycetes*. However, in the intestinal microbiota of CL *A. fabalis*, the positive correlation between *Firmicutes* and *Actinomycetes* is the main network relationship, and there are also scattered connections between a small number of *Firmicutes*. Compared with SL, the correlation network of CL *A. fabalis* flora is closer. Diet may be one of the important driving factors for the composition of intestinal microbiota of birds in each lake. The obvious difference in the composition of intestinal microbiota may also reflect a high sensitivity of intestinal microbiota to the food sources of the two lakes [16]. The microbial correlation network analysis in this study also provides a further research basis for researchers to construct in vitro microbial networks in the future.

In this study, we used PICRUSt1 to predict and analyze the COG functional abundance of bacterial 16S rDNA sequences. It was found that there was no significant difference in the relative abundance of COGs annotated based on SL and CL samples (*p* < 0.05), mainly including energy production and transformation, amino acid transport and metabolism, carbohydrate transport and metabolism, and transcription (*p* < 0.05). These functions are closely related to the structure of intestinal microbiota. This result is similar to the result predicted based on the intestinal microbiota community of Poyang Lake Swan [30]. By studying the composition and structural characteristics of the intestinal microbiota of migratory *A. fabalis*, we provide basic data for the study of intestinal microbiota of *A. fabalis*.

Through analysis of the intestinal microbiota of *A. fabalis*, we found that Firmicutes is the dominant phylum and *Lactobacillus* is the main genus in the intestinal microbiota of SL and CL *A. fabalis*. In the intestinal microbiota of SL *A. fabalis*, there is only a positive correlation between *Bacteroidetes* and *Proteobacteria*, and there is a close relationship between species. At the same time, there are positive and negative correlations between *Firmicutes* and *Actinomycetes*. In CL *A. fabalis*, the positive correlation between *Firmicutes* and *Actinomycetes* is the main network relationship, and there are also a small number of connections between *Firmicutes*. It is found that the COG functions of SL and CL bacteria mainly include energy production and transformation, amino acid transport and metabolism, carbohydrate transport and metabolism, and transcription. This study provides basic data for the study of intestinal microbiota of *A. fabalis*. Of course, this study also has some limitations. Because migratory birds arrive in November every year and leave in March the following year, we cannot explore long-term and different seasonal changes. At the same time, because migratory birds are rare and protected animals, they cannot be caught and studied privately. At present, our research team is actively contacting relevant departments to apply for authorization documents for migratory bird research on the premise of protecting migratory birds as much as possible, so as to provide more and more scientific basic data for migratory bird research.

## 5. Conclusions

*Lactobacillus* is the main genus of *A. fabalis*, of which *Lactobacillus aviaries* is the highest. There was only a positive correlation between *Bacteroidetes* and *Proteobacteria* in the intestinal microbiota of SL *A. fabalis*, and the species were closely related. However, CL is mainly related to the positive correlation between *Firmicutes* and *Actinomycetes.* The functions of intestinal microbiota of *A. fabalis* mainly include energy production and transformation, amino acid transport and metabolism, carbohydrate transport and metabolism, and transcription. This study provides basic data for the study of intestinal microbiota of *A. fabalis*.

## Figures and Tables

**Table 1:** Information form of *Anser fabalis* fecal sample collection.

Sampling Period	Species	Sampling Place	Sampling Habitat	Number
10 November 2021–4 March 2022	*Anser fabalis*	Yang etou	vast expanse of grassland at waterside	60
16 November 2021–12 March 2022	*Anser fabalis*	Caizi Lake	vast expanse of grassland at waterside	60

## Data Availability

The data that support the findings of this study are available from the corresponding author upon reasonable request.
